# Increased metallopeptidase activity, compromised endothelial integrity and impaired pathogen recognition revealed by transcriptomics in *Angiostrongylus vasorum*-infected dogs with hypocoagulability and vascular dysfunction

**DOI:** 10.1016/j.crpvbd.2026.100378

**Published:** 2026-04-12

**Authors:** Lucienne Tritten, Annageldi Tayyrov, Lennart Opitz, Annette P.N. Kutter, Nadja E. Sigrist, Natalie Hofer-Inteeworn, Claudia Kümmerle-Fraune, Manuela Schnyder

**Affiliations:** a Institute of Parasitology, Vetsuisse Faculty, University of Zurich, Winterthurerstrasse 266a, Zürich, 8057, Switzerland; b Functional Genomics Center Zurich, ETH Zurich/University of Zurich, Winterthurerstrasse 190, Zurich, 8057, Switzerland; c Section of Anaesthesiology, Department of Clinical Diagnostics and Services, Vetsuisse Faculty, University of Zurich, Winterthurerstrasse 260, Zurich, 8057, Switzerland; d Division of Emergency and Critical Care Medicine, Department of Small Animals, Vetsuisse Faculty, University of Zurich, Winterthurerstrasse 258c, Zurich, 8057, Switzerland; e Clinic for Small Animal Internal Medicine, Department of Small Animals, Vetsuisse Faculty, University of Zurich, Zurich, Switzerland

**Keywords:** *Angiostrongylus vasorum*, Dog, Transcriptome, Consumptive coagulopathy, Vascular endothelium disfunction, Inflammation, Impaired fibrinogen-dependent clotting

## Abstract

Dogs infected with the cardiopulmonary nematode *Angiostrongylus vasorum* are at high risk to die because of the associated bleeding pathologies, which remain poorly understood. Given their central role in immune surveillance and signaling, we analyzed transcriptomic profiles from (i) *A. vasorum*-infected dogs presenting with hypocoagulability (Inf_hypo_; *n* = 5), (ii) infected dogs with normal coagulation values (Inf_norm_; *n* = 5), and (iii) uninfected controls (UC; *n* = 12). Alongside classical hematology and coagulation parameters, including rotational thromboelastometry (ROTEM), we generated a global transcriptomic profile of circulating blood cells, enabling the identification of differentially expressed genes following infection. Fibrinogen levels were significantly lower in Inf_hypo_ than in Inf_norm_ dogs. All Inf_hypo_ dogs showed out-of-range prothrombin times. Matrix metalloprotease transcripts were strongly upregulated among infected dogs, regardless of their coagulation status, which might amplify vascular disruption and inflammation. Analysis of downregulated transcripts in infected dogs compared to UC indicated a loss of endothelial integrity, especially at the level of the lungs, and of inflammatory processes contributing to fibrinolysis and coagulopathies. Endothelial injury is a known trigger of disseminated intravascular coagulation (DIC) and consumption of coagulation factors, which could explain the observed coagulation disorders induced by *A. vasorum* infection. The multifaceted alterations observed in hematologic and coagulation parameters, ROTEM, and transcriptomic profiles of *A. vasorum*-infected dogs point to a dynamic and evolving disruption of hemostatic balance. While some animals show signs of early compensatory hypercoagulability, others seem to progress toward a state consistent with DIC, marked by factor consumption, endothelial dysfunction, and impaired fibrinogen-dependent clotting.

## Highlights

• *Angiostrongylus vasorum* is a potentially lethal cardiopulmonary nematode of dogs.
• We conducted a global blood transcriptomics analysis in *A. vasorum*-infected dogs.
• Hypocoagulable dogs showed low fibrinogen and altered prothrombin times.
• Upregulated metalloproteinases are associated with vascular damage and inflammation.
• Data suggest coagulopathy progressing from compensation to fibrinolysis and disseminated intravascular coagulation.

## Graphical abstract

**Figure:**
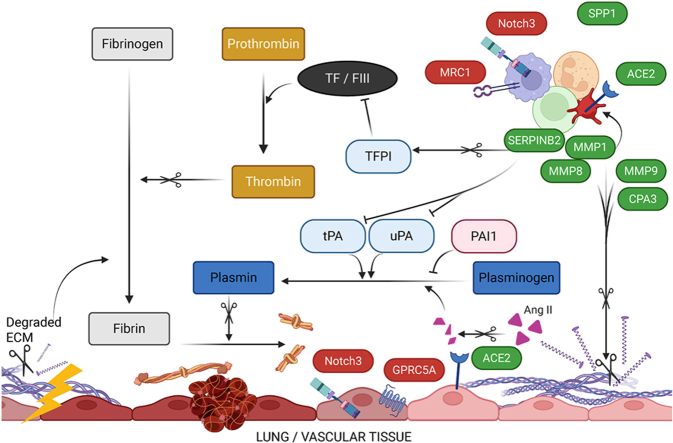

## 1 Background

*Angiostrongylus vasorum*, also known as the French heartworm, is a nematode parasite of wild carnivores such as red foxes, and of domestic dogs, which can lead to fatal outcomes. In these definitive hosts, adult worms reside in the pulmonary arteries and the right ventricle of the heart (Rosen et al., 1970), where they release numerous eggs. In the past decades, *A. vasorum* has been spreading rapidly among wild carnivores in several European countries (Taylor et al., 2015; Traversa et al., 2019; Gillis-Germitsch et al., 2020; Lemming et al., 2020). The dramatic rise in prevalence among foxes, i.e. from approximately 20% in 2012 to 82% by 2017 in Switzerland, highlights the rapid spread of *A. vasorum* and underscores the role of foxes as wildlife reservoirs in Europe (Gillis-Germitsch et al., 2020).

Prevalence in domestic dogs lies between 0.5 and 3% (Morgan et al., 2021). If left untreated, this infection is linked to a high fatality rate, with approximately two-thirds of naturally infected animals at risk of succumbing to the illness (Staebler et al., 2005). Dogs with *A. vasorum* infections may remain subclinical for some time, or they may present with a diverse range of clinical signs. Respiratory disease and bleeding disorders are common, accompanied by a wide range of other, often nonspecific, clinical signs (Chapman et al., 2004; Koch and Willesen, 2009). Hence, timely identification poses a notable challenge and has to be supported by suitable diagnostic tools (Schnyder et al., 2014, 2015).

Unexplained bleeding may represent the primary presenting complaint in dogs infected with *A. vasorum* (Glaus et al., 2016). Bleeding disorders have been reported in 15–35% of infected dogs (Koch and Willesen, 2009; Morgan et al., 2010; Becskei et al., 2020), with overt bleeding tendencies observed in approximately one-third of cases (Adamantos et al., 2015). Clinical manifestations range from petechiae and hematomas to internal hemorrhage and secondary neurological dysfunctions (Cury and Lima, 1996; Garosi et al., 2005; Wessmann et al., 2006; Sasanelli et al., 2008; Denk et al., 2009; Sigrist et al., 2017). Dogs with coagulation deficiencies may be at higher risk of fatal outcomes (Staebler et al., 2005; Adamantos et al., 2015). Despite several proposed hypotheses, the underlying mechanisms driving these coagulopathies remain poorly understood. Suggested causes include deficiencies in coagulation factors, e.g. FV and FVIII, immune-mediated thrombocytopenia, secretion of anticoagulants by the parasite, hyperfibrinolysis (HFL) (Zoia and Caldin, 2015; Sigrist et al., 2017, 2021), and (chronic) disseminated intravascular coagulation (DIC) (Schelling et al., 1986; Caruso and Prestwood, 1988; Ramsey et al., 1996; Cury et al., 2002; Koch and Willesen, 2009; Adamantos et al., 2015; Zoia and Caldin, 2015).

Recently, HFL, along with concurrent hypofibrinogenemia, was identified in 67% of *A. vasorum*-infected dogs presenting with clinical signs of bleeding (Sigrist et al., 2017). In a prospective follow-up study, rotational thromboelastometry (ROTEM) revealed a notable decrease in clot stability among dogs exhibiting overt signs of bleeding tendency (Sigrist et al., 2021). A quantitative proteome analysis of sera from dogs experimentally infected with the parasite revealed potentially impaired biological pathways, notably the complement system (especially the lectin pathway) and the coagulation cascade. This inference was based on the observation of significantly reduced levels of several key proteins in infected dogs (Tritten et al., 2021). Notably, levels of mannan-binding lectin, serine peptidases, ficolin, and coagulation factor XIII-B were lower compared to pre-infection values. In addition, soluble proteins released by adult worms contain putative modulators of host coagulation. While these proteins may not be solely responsible for bleeding diatheses, they could contribute to their development (Gillis-Germitsch et al., 2021a). Overall, our understanding of the pathomechanisms underlying canine angiostrongylosis has often been gleaned from isolated case studies lacking a systematic descriptive approach. The existing body of knowledge remains insufficient for a comprehensive understanding and evidence-based management of coagulopathies associated with *A. vasorum*, and multiple hypotheses remain plausible and merit further investigation.

Comparative transcriptome analysis has been instrumental in offering deep insights into gene regulatory networks, biological pathways, and infection biomarkers that undergo alterations during helminth infections (Zhou et al., 2016). Blood may accurately reflect immune responses to an infection or a pathophysiological condition. For instance, the transcriptomic profile of canine peripheral blood nuclear cells has previously facilitated the identification of two signaling pathways implicated in animals with heart failure (Hulanicka et al., 2014). Following a similar approach, researchers identified the immune mechanisms triggered by *Mycobacterium bovis* infection in cattle by analyzing mixed peripheral blood leukocytes (Killick et al., 2011; McLoughlin et al., 2014). Collectively, transcriptomic studies have been invaluable in advancing our comprehension of diseases and host-parasite interactions (Li et al., 2010; Sutherland et al., 2011; Hulanicka et al., 2014).

Since immunological aspects are highly relevant for infectious diseases and blood is also “the pipeline of the immune system” (Chaussabel et al., 2010), we performed a comparative analysis of transcriptomic profiles of blood from: (i) naturally infected dogs with *A. vasorum* presenting with hypocoagulability, (ii) infected dogs exhibiting normal coagulation parameters, and (iii) uninfected animals. We generated a comprehensive blood cell transcriptomic profile to identify differentially expressed genes following infection and varying states of coagulation with the aim to provide valuable insights into the intricate interplay between infection, coagulation, and immune responses.

## 2 Materials and methods

### 2.1 Animals

Dogs across all breeds, ages and sexes were eligible for the present study. Dogs were excluded if they were pre-treated with antifibrinolytics, blood products, tranexamic acid, steroids and/or NSAIDS within four weeks of presentation or were diagnosed with another disease. Eight animals were naturally infected and presented to the Animal Hospital of the University of Zurich between December 2015 and December 2018. Two additional animals were experimentally infected with 100 third-stage larvae of *A. vasorum* in a research facility in the frame of other studies. *Angiostrongylus vasorum-*positive dogs were diagnosed by either serological antigen ELISA or rapid-assay (AngioDetect™, IDEXX Laboratories, Westbrook, Maine, USA) (Schnyder et al., 2014, 2015), and/or copromicroscopic identification of first-stage larvae in feces (Baermann funnel method) (Deplazes et al., 2016). Twelve healthy healthy dogs representing the uninfected control group had normal complete blood cell profiles, plasmatic coagulation times and ROTEM analysis and tested negative for *A. vasorum* infection.

### 2.2 Sample acquisition, hematology and coagulation parameters

Blood was drawn at a limb using a 20 G needle to fill the following tubes, always respecting the same order: PAXgene Blood RNA Tube (BD Biosciences), citrate, and EDTA tubes. The PAXgene tubes were frozen at −80 °C until RNA isolation; the citrate and EDTA tubes were brought immediately to the hospital’s laboratory for the following automated analyses: plasmatic coagulation (Start 4, STAGO CH SA, Glattbrugg, Switzerland); ROTEM (TEM International GmbH, Munich, Germany); and complete blood count (Sysmex-XT, 2000iV, Sysmex Cooperation, Kobe, Japan), including cell differentiation. The following coagulation parameters were assessed: prothrombin time (PT; s); partial thromboplastin time (PTT; s); thrombin time (TT; s), fibrinogen (g/l) measured by Clauss and, when possible (*n* = 16), D-dimer concentrations (mg/l). ROTEM analysis included Fib-TEM (tissue factor activated ROTEM with inhibition of thrombocyte function), In-TEM (intrinsically activated ROTEM), and Ex-TEM (tissue factor activated ROTEM; all Axon Lab, Baden, Switzerland), Maximum Clot Firmness (MCF), and Maximum Clot Elasticity (MCE), performed as described elsewhere (Sigrist et al., 2017, 2021).

Statistical analysis was performed using R studio (R version 4.4.2). Variance analysis of mean coagulation, ROTEM and hematology parameters was conducted using the Kruskal-Wallis and Dunn’s *post-hoc* test with Bonferroni correction.

### 2.3 Determination of coagulation status and cohort classification based on hematology and coagulation parameters

Interpretation of all coagulation data was performed in a blinded manner with respect to infection status. Classification of *A. vasorum-*positive dogs into hypocoagulable (Inf_hypo_) or normocoagulable (Inf_norm_) was guided by expert consensus (APNK, NES). The dogs were classified as hypocoagulable if two or more of the following alterations were seen: prolonged PT or Ex-TEM-clotting time (CT); prolonged PTT or In-TEM-CT; decreased fibrinogen or Fib-TEM-maximum clot firmness (MCF); decreased Ex-TEM or In-TEM MCF; Maximum lysis > 14%; or Ap-TEM-MCF > Ex-TEM-MCF (Supplementary Table S1). All processes have been validated at the Veterinary Hospital of the University of Zurich, and the analyses followed standard procedures.

### 2.4 Transcriptome analysis

RNA was isolated from blood (PAXgene tubes) with the PAXgene Blood RNA kit IVD (PreAnalytiX, Hombrechtikon, Switzerland), followed by DNase treatment. RNA quantification was carried out using fluorometry (Qubit 1.0). Sample quality was assessed with a fragment analyzer (Agilent Technologies, Santa Clara, CA, USA) at the Functional Genomics Center Zurich, ensuring a minimal RNA Integrity Number (RIN) of 7.7 for further processing. For library preparation and sequencing, samples were processed using the Illumina TruSeq mRNA library protocol (TruSeq RNA kit, polyA selection). RNA sequencing was performed on an Illumina Novaseq 6000 sequencer, generating 100 bp single-end reads. Quality control of raw sequencing data was performed using FastQC, and potential contaminations were checked with FastQScreen.

Sequencing reads were aligned using STAR (Spliced Transcripts Alignment to a Reference) (Dobin et al., 2013) with the Ensembl dog genome build (*Canis familiaris* v.3.1) as reference. Transcript quantification was conducted with Kallisto (Bray et al., 2016). Trimmed mean of M-values (TMM) was used to normalize read counts, and differential expression was tested with the quasi-likelihood (QL) test. For pairwise comparison of differentially expressed (DE) genes between infected animals and uninfected controls (with further stratification based on coagulation status), we used the Bioconductor package *EdgeR*. Gene set enrichment analysis (GSEA) was computed with the *clusterProfiler* package. To complement this, the online version of Enrichr was employed in some instances for pathway analysis (Kuleshov et al., 2016), based on log2 fold-change (FC) cut-off of 1.0.

## 3 Results

A total of 10 dogs infected with *A*. *vasorum* and 12 uninfected controls (UC) were included in the study. All included dogs were part of concurrent studies (Jud Schefer et al., 2020; Sigrist et al., 2021). The stratification of infected dogs into two groups based on their coagulation status, normocoagulable (Inf_norm_, *n* = 5) and hypocoagulable (Inf_hypo_, *n* = 5), enabled the investigation of differences in laboratory findings between the groups and how variations in host coagulation status influence transcriptomic responses to infection.

### 3.1 Hematology and coagulation

A complete cell blood count was established for each dog, including cell differentiation (automatic or manual: Supplementary Table S1 and Supplementary Fig. S1). Basophil counts were significantly higher in infected dogs compared to UC (adj. *P* < 0.001 for both infected groups compared to UC). Eosinophil counts were higher in Inf_norm_ compared to UC (adj. *P* = 0.03); a trend was observed in Inf_hypo_ as well, without reaching statistical significance (adj. *P* = 0.6). Similarly, leukocyte and neutrophil counts were only slightly elevated in infected animals and compared to UC (adj. *P* > 0.05). Monocyte and lymphocyte counts were not different across groups. Thrombocyte counts were slightly lower in Inf_hypo_, compared to both Inf_norm_ and UC, however, not significantly different (both adj. *P* = 0.1, Supplementary Fig. S1). In line with this, the thrombocrit (%) was lower in the 3 Inf_hypo_ animals for which these data were available, compared to both other groups (adj. *P* = 0.1, and 0.3, respectively), while thrombocyte volume (fl) was lower in all infected subjects compared to UC (both with adj. *P* > 0.3).

Mean PT was 9.5 s (reference interval 6.5–8.5 s) in infected animals compared to 7.1 s in UC (*P* = 0.048). PTT, PT, and TT were all significantly prolonged in infected dogs (Inf_norm_ and Inf_hypo_ taken together) compared to UC. A significantly elevated mean PTT of 13.8 s was measured in *A. vasorum-*infected dogs, compared to 11.6 s in UC (*P* = 0.045). In both groups, values were still within the reference intervals. Similarly, mean TT was increased by 3 s in infected animals to 16.7 s, compared to 13.7 s in the absence of infection (*P* = 0.046), both within the reference interval (12.3–21.6 s). Fibrinogen was not different upon infection compared to UC.

**Fig. 1:**
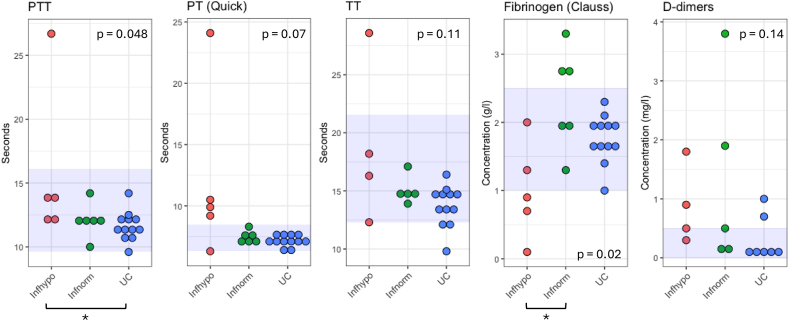
Coagulation test results. PTT, PT (Quick) and TT results are expressed in seconds. Fibrinogen concentration (by Clauss) is expressed in g/l and D-dimer concentration values, available for some samples only, in mg/l. Reference intervals established for dogs were determined by the laboratory of the Animal Hospital of the University of Zurich and are delimited by blue zones: 9.6–16.1 s for PTT, 6.3–8.5 s for PT (Quick), 12.3–21.6 s for TT, 1.0–2.5 g/l for fibrinogen (Clauss), and ≤ 0.5 mg/l for D-dimer concentrations. Variance analysis of mean coagulation parameters was conducted using the Kruskal-Wallis test and Dunn’s *post-hoc* test with Bonferroni correction for pairwise comparisons allowed to identify which groups differ from each other. ∗adj. *P* < 0.05. *Abbreviations*: PTT, partial thromboplastin time; PT (Quick), prothrombin time; TT, thrombin time; Inf_hypo_, *A. vasorum-*positive dogs into hypocoagulable; Inf_norm_, *A. vasorum-*positive dogs into normocoagulable; UC, uninfected controls.

When stratifying the *A. vasorum* infected group according to the coagulation status, significant differences appeared (Fig. 1 and Supplementary Table S1). PTT was slightly longer in Inf_hypo_ compared to UC (*P* = 0.042). PT was not significantly different between Inf_hypo_ and UC (adj. *P* = 0.067), despite most individual PT values obtained for the Inf_hypo_ group being outside of the reference range. The mean fibrinogen concentration was decreased (*P* = 0.02) in the Inf_hypo_ group (1.0 g/l) but increased in Inf_norm_ (2.33 g/l) compared to UC (1.78 g/l). Accordingly, fibrinogen showed the most pronounced differences across groups, but all values lied within the reference interval (1.0–2.5 g/l). D-dimer concentrations were out of the reference interval in 4 out of 8 infected animals regardless of coagulation status (0.88 mg/l in Inf_hypo_ and 1.30 mg/l in Inf_norm_), while in 2 out of 7 were also higher than reference intervals in UC. No difference across groups could be found (*P* = 0.14).

ROTEM analysis revealed distinct coagulation profiles across groups (Table 1). Dogs in the Inf_hypo_ group showed significantly prolonged clotting times (CT), compared to both Inf_norm_ (CT _Ex-tem_: 29 s, CT _In-tem_: 161 s) and UC (CT _Ex-tem_: 34 s, CT _In-tem_: 155 s). Clot formation times (CFT) were also markedly longer in Inf_hypo_ dogs. Inf_hypo_ animals had lower maximum clot firmness (MCF) across multiple assays: in Ex-tem and In-tem, median MCF were 39 mm and 40.5 mm, respectively, *versus* 59 mm and 63.5 mm in the Inf_norm_ group and 49 mm and 59 mm in UC. This was accompanied by lower alpha angles in Inf_hypo_ (Ex-tem: 41°, In-tem: 51.5°), reflecting slower clot kinetics. Fibrinogen-dependent clotting (Fib-tem) was almost absent in Inf_hypo_ (MCF: 0 mm), whereas both Inf_norm_ (7 mm) and UC (4 mm) showed normal fibrin contribution. In contrast, the Inf_norm_ group displayed coagulation parameters within or above reference ranges, suggesting normo- to hypercoagulable status. Their MCF and MCE values were the highest across all assays, and alpha angles indicated rapid clot formation. For instance, MCE _In-tem_ reached a median of 172 compared to 67 in Inf_hypo_ and 146 in UC. The UC group presented intermediate coagulation profiles, with most values falling between Inf_hypo_ and Inf_norm_. Their clotting times and MCF were within reference intervals, though variability was greater, especially in parameters like CT _Ex-tem_ (range: 25–94 s) and MCE _Ex-tem_ (29–197 mm).

**Table 1:** Rotational thromboelastometry (ROTEM) parameters across groups with a different coagulation status.

Parameter	Inf_hypo_			Inf_norm_			UC			Reference interval (Sigrist et al., 2021)	*P*-value
	*n*/*N*	Median	Range	*n*/*N*	Median	Range	*n*/*N*	Median	Range		
CT_Ex-tem_ (s)	5/5	113	35–251	5/6	29	26–60	11/12	34	25–94	23–87	0.01∗∗
CFT_Ex-tem_ (s)	4/5	390	287–427	5/6	114	77–395	11/12	201	82–1306	85–357	0.07
Alpha_Ex-tem_ (°C)	5/5	41	18–47	5/6	75	47–77	11/12	58	23–74	42–77	0.01∗∗
MCF_Ex-tem_ (mm)	5/5	39	14–45	5/6	59	35–65	11/12	49	23–66	32–65	0.049∗
MCE_Ex-tem_ (mm)	5/5	55	17–83	5/6	146	53–189	11/12	96	29–197	45–142	0.08
ML_Ex-tem_ (%)	5/5	3	0–99	5/6	2	0–6	11/12	6	1–86	0–12	0.32
CT_In-tem_ (s)	4/5	228.5	158–315	4/6	161	146–233	11/12	155	116–206	133–210	0.15
CFT_In-tem_ (s)	4/5	223	1–294	4/6	91	66–105	11/12	97	59–121	59–201	0.28
Alpha_In-tem_ (°C)	4/5	51.5	20–64	4/6	73	69–76	11/12	71	67–78	58–78	<0.01∗∗
MCF_In-tem_ (mm)	4/5	40.5	30–49	4/6	63.5	57–66	11/12	59	57–68	52–71	<0.01∗∗
MCE_In-tem_ (mm)	3/5	67	42–96	3/6	172	134–181	10/12	146	130–211	108–242	0.03
ML_In-tem_ (%)	4/5	1	0–80	4/6	0	0–2	11/12	1	0–79	0–3	0.4
CT_Fib-tem_ (s)	5/5	3.6	3.6–39	4/6	36	35–53	11/12	35	22–464	21–112	0.04∗
MCF_Fib-tem_ (mm)	4/5	0	0–4	4/6	7	4–10	11/12	4	2–8	2–9	0.02∗
MCE_Fib-tem_ (mm)	1/5	4	4–4	4/6	7.5	4–12	11/12	4	2–9	2–9	0.22

*Note*: *P*-values determined by Kruskal-Wallis test; ∗*P* < 0.05, ∗∗*P* ≤ 0.01.

*Abbreviations*: CT, clotting time; CFT, clot formation time; Ex-tem, tissue factor activated ROTEM; Fib-tem, tissue factor activated ROTEM with inhibition of thrombocyte function; In-tem, intrinsically activated ROTEM; MCF, maximum clot firmness; ML, maximum lysis; *n*, number of dogs analyzed; *N*, number of dogs in group; PT, prothrombin time; MCE, maximum clot elasticity. Inf_hypo_, infected, hypocoagulable; Inf_norm_, infected, normocoagulable; UC, uninfected controls.

### 3.2 Transcriptomic profiles reflect infection and coagulation status

**Fig. 2:**
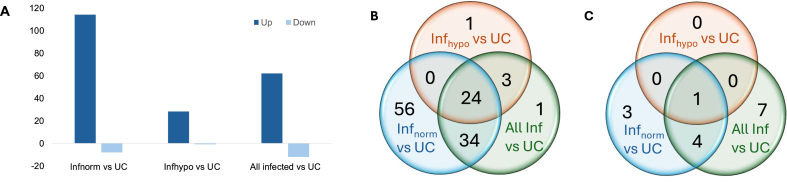
Differential gene expression analysis. Four groups were compared pairwise (UC, All Inf, Inf_norm_, Inf_hypo_; FDR = 0.05, log2 FC > 1). **A** Overview of the number of differentially expressed genes in pairwise comparisons. **B** Venn diagram showing upregulated transcripts with detailed gene symbol lists. **C** Venn diagram showing downregulated transcripts with detailed gene symbol lists. *Abbreviations*: Inf, infected; Inf_hypo_, *A. vasorum-*positive dogs, hypocoagulable; Inf_norm_, *A. vasorum-*positive dogs, normocoagulable; UC, uninfected controls.

**Fig. 3:**
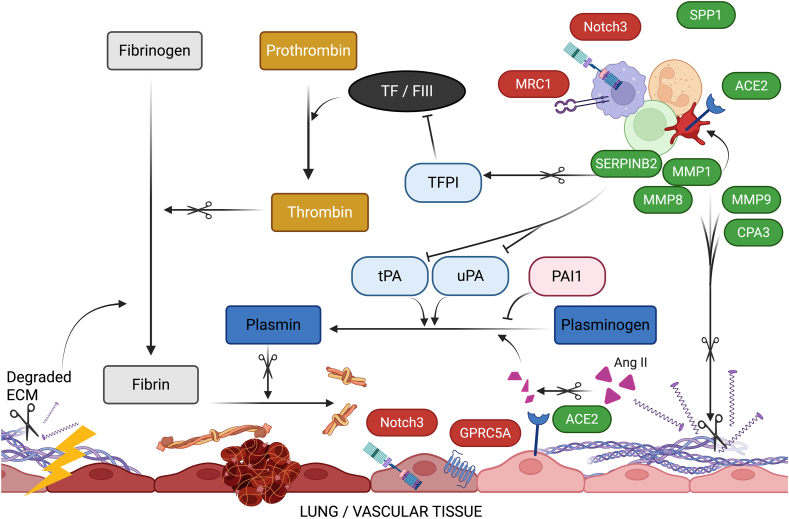
Functional network overlay of dysregulated genes across vascular, coagulation, and degradation pathways. This schematic integrates transcriptionally dysregulated genes into a functional network encompassing vascular homeostasis, coagulation, fibrinolysis, and extracellular matrix (ECM) remodeling. *Green* and *red* indicate up- and downregulation, respectively, in infected animals *versus* uninfected controls. Nodes represent significantly dysregulated genes; arrows indicate interactions, regulatory effects or proteolytic activity. Key regulators are positioned according to their roles in thrombin generation (e.g. TF/FIII, Thrombin), fibrin formation and degradation (Fibrin, Plasmin), fibrinolsis (PAI1, SERPINB2, uPA, tPA), and matrix remodeling (MMP1, MMP8, MMP9, CPA3). Transcript changes in *ACE2*, *Notch3*, *MRC1*, *GPRC5A*, and *SPP1* highlight vascular signaling and immune-epithelial cross-talk. MMP1 and MMP8, released by endothelial and immune cells, promote coagulation *via* ECM collagen degradation (Amar et al., 2017), enabling platelet activation and TFPI cleavage (Belaaouaj et al., 2000). MMP9, a gelatinase with some collagenase activity, contributes to ECM degradation and endothelial injury (lightning bolt), further triggering coagulation, including *via* TF. SERPINB2 (PAI-2), produced by macrophages and bronchial epithelium, inhibits fibrinolysis through tPA and uPA, stabilizing clots (Medcalf, 2011). ACE2 regulates the renin-angiotensin system and maintains alveolar-capillary integrity (Imai et al., 2005; Kuba et al., 2005); elevated *ACE2* mRNA in blood reflects epithelial and endothelial injury, including sepsis (Zhang et al., 2020; Jalaleddine et al., 2023; Li et al., 2024). ACE2 also exerts antithrombotic effects by degrading Angiotensin II (Ang2) and limiting PAI-1 induction (not shown). CPA3, a mast cell protease, cooperates with MMP9 in collagen degradation, increases vascular permeability, and is associated with pulmonary fibrosis in the context of lung injury (Meneses-Preza et al., 2024). SPP1 is linked to pulmonary thromboembolism (Lu et al., 2022), vascular calcification, and inflammation (Jung et al., 2025; Zhao et al., 2024). Notch3 contributes to vascular integrity and may indirectly modulate coagulation (Romay et al., 2024). GPRC5A supports epithelial homeostasis, and its loss predisposes to lung injury (Liao et al., 2015). MRC1 (*CD206*), expressed by alternatively activated macrophages, promotes resolution of inflammation (Reyfman et al., 2019; Schyns et al., 2019). Figure generated in BioRender.

Illumina sequencing produced between 17.8 and 54 million raw reads per sample, which were aligned to the dog genome; at least 97% mapped to the genome in each sample. Differential gene expression (DGE) analysis was performed in pairwise group comparisons across uninfected control animals and *A. vasorum-*infected dogs with different coagulation status. Sixty-two transcripts were found to be upregulated with a fold-change (FC) > 2 (FDR = 0.05) in infected animals compared to uninfected controls (UC), whereas 12 transcripts appeared at reduced levels upon infection (Fig. 2A). The analysis was further stratified according to coagulation status; most upregulated transcripts appeared when Inf_norm_ animals were compared to UC (114 up- and 8 downregulated). Surprisingly, transcript expression did not differ significantly between infected dogs with different coagulation status using the same FC and FDR cut-offs (no up- or downregulated genes to report). A detailed list of up- and downregulated genes in each pairwise comparison is shown in Fig. 2B and C, as well as in Supplementary Tables S2–S5. Twenty-four genes were consistently upregulated in all pairwise comparisons between infected animals and UC. These were particularly enriched in enzymes with metallopeptidase activity. Our results are presented in a broader context in Fig. 3, linking coagulation, immune responses, and endothelial health.

#### 3.2.1 Inf_norm_*vs* UC dogs

A total of 114 genes were upregulated (Fig. 2B) compared to UC, including *MMP1* (log2 FC = 8.17), and others with metallopeptidase activity. Among the 8 downregulated genes (Fig. 2C) resulting from the comparison between Inf_norm_ dogs and UC (*MRC1*, *FARP1*, *GPRC5A*, *STYXL2*, *NOTCH3*, *SCAMP5*, *PTPRS*, and *CYP2D15*), several encode proteins which maintain endothelial integrity (Notch3, FARP1, and GPCR5A) or are involved in pathogen recognition (MRC1, PTPRS, and STYXL2).

#### 3.2.2 Inf_hypo_*vs* UC dogs

Twenty-eight genes were upregulated in Inf_hypo_ animals compared to UC (Fig. 2B). *MMP1*, *MMP9*, *ACE2*, and *CPA3* showed log2 FC > 5 in this comparison, and *MMP8* showed a log2 FC of 3.24. Most importantly, *MMP1* showed a log2 FC of 8.47. Pathway analysis (MSigDB *via* Enrichr) revealed the ‘*coagulation*’ as the most enriched pathway (adj. *P* = 0.007), contributed by upregulation of matrix metalloproteases 1 and 9 (log2 FC = 8.47 and 4.59, respectively), and the plasminogen activator inhibitor-2 (*SERPINB2*; log2 FC = 2.24), followed by ‘*angiogenesis*’ (adj. *P* = 0.007), contributed by secreted phosphoprotein 1 (*SPP1*, or osteopontin; log2 FC = 1.65) and peptidoglycan recognition protein 1 (*PGLYRP1*; log2 FC = 2.53). In this comparison, only one gene, *PTPRS*, was found to be significantly downregulated, while two additional significantly downregulated genes were slightly below the log2 FC cut-off of 1, which we applied (−0.92 and −0.97, respectively): aryl hydrocarbon receptor repressor (AHRR), and tyrosine kinase with immunoglobulin and EGF homology domains (TIE2, encoded by the gene *TEK*). AHRR is a regulator of inflammation and a potent tumor suppressor protein, and its downregulation is observed in various human tumoral tissues (including the lung) (Vogel and Haarmann-Stemmann, 2017). TIE2 is a receptor for angiopoietins (Angpt), mainly expressed by endothelial cells and is associated with vascular health.

#### 3.2.3 Pathway enrichment

Gene set enrichment analysis (GSEA) was compiled based on all identified transcripts to provide a functional dimension to the obtained data (Supplementary Tables S6–S8). Six Gene Ontology (GO) ‘biological process’ pathways differed comparing Inf_hypo_ to Inf_norm_: ‘innate immune response’ (GO:0045087) and ‘immune system processes’ (GO:0002376) were significantly negatively enriched (hence, weakened) in Inf_hypo_ dogs compared to Inf_norm_. The other four significantly negatively enriched pathways in Inf_hypo_ dogs were within defense processes against viruses and bacteria. Among GO ‘biological processes’, translation (GO: 0006412) is consistently increased upon *A. vasorum* infection (stratified by coagulation status or not) compared to UC. Similarly, the GO ‘molecular functions’ metallopeptidase activity (GO: 0008237) is enhanced across all infection groups. In Inf_hypo_ compared to UC, endopeptidase activity (GO: 0004175) increased as well.

## 4 Discussion

Coagulopathies in dogs with *A. vasorum* infection present a clinical and diagnostic challenge. In a previous study, only two-thirds of hypocoagulable dogs showed bleeding, while over a quarter of non-bleeding dogs were hypocoagulable and thus at risk of deterioration during hospitalization (Sigrist et al., 2021). This underscores the complexity of clinical presentations associated with *A. vasorum* infection (Willesen et al., 2022). Even among animals with measurable hemostatic dysfunction, the manifestation of clinical signs is highly variable.

Hemostatic abnormalities are well recognized in *A. vasorum* infection. In one study of 20 naturally infected dogs, eosinophilia, leukocytosis, monocytosis, lymphocytosis, and basophilia were observed with decreasing frequency (Chapman et al., 2004), although the reported prevalence of these alterations varies across studies (Cury et al., 2002; Schnyder et al., 2010; Willesen et al., 2009, 2022). In our cohort, infected dogs showed significantly higher basophil counts than uninfected controls (UC). While basophilia is rare in dogs and difficult to assess *via* blood smears, it is associated with hypersensitivity reactions and inflammatory responses (Schulze, 2000). Eosinophilia - commonly linked to helminth migration and IL-5 activity (Schulze, 2000) - was detected in the Inf_norm_ group, although this finding is not consistently reported in *A. vasorum* infections (Cury et al., 2002; Chapman et al., 2004; Willesen et al., 2009, 2022). Neutrophil counts in our study differed minimally between infected and control dogs, consistent with earlier reports of mild and transient neutrophilia (Cury et al., 2002; Chapman et al., 2004; Willesen et al., 2022), particularly following anthelmintic treatment (Schnyder et al., 2010). Mild thrombocytopenia, frequently observed in infected dogs (Ramsey et al., 1996; Chapman et al., 2004; Sigrist et al., 2018; Willesen et al., 2022), often emerges near patency (Schnyder et al., 2010), and our data (although limited to a small sample size) suggest it is likely linked to hypocoagulability. To further characterize these effects at the functional level of coagulation, we examined clot formation dynamics using ROTEM analysis. ROTEM further substantiated the presence of distinct coagulation phenotypes across groups. Strikingly, fibrinogen-dependent clot formation was nearly absent in Inf_hypo_ dogs, whereas it was preserved in both Inf_norm_ and UC dogs. The coagulation and ROTEM results confirm a pronounced deficit in both cellular and plasma-based contributors to coagulation in the Inf_hypo_ group. The UC group exhibited intermediate profiles, with values generally falling between those of Inf_hypo_ and Inf_norm_ dogs. While most UC parameters were within reference intervals, several showed broad variability, possibly reflecting subclinical variability or co-factors unrelated to *A. vasorum* infection. Together, these hematological alterations suggest that *A. vasorum* infection affects both inflammatory and hemostatic pathways.

Retrospective analyses have shown that prolonged prothrombin time (PT) and partial thromboplastin time (PTT) are common, though inconsistently reported, in bleeding dogs with *A. vasorum* infection (Chapman et al., 2004), with more recent studies confirming this trend (Adamantos et al., 2015). PT and PTT assess the integrity of the intrinsic, extrinsic and common coagulation pathways and may become prolonged when coagulation factors involved in these cascades, including factors XII, XI, IX, VIII, X, V, prothrombin and fibrinogen, are reduced or dysfunctional (Raber, 1990; Wheeler et al., 2022). While our sample size limits definitive conclusions, we observed a tendency toward prolonged PTT and a near-significant extension of PT in the Inf_hypo_ group. Notably, all Inf_hypo_ dogs showed PT values outside the normal range, four prolonged and one shortened. Fibrinogen concentrations were significantly lower in Inf_hypo_ animals compared to Inf_norm_. Hypofibrinogenemia is frequently reported in *A. vasorum*-infected bleeding dogs (Adamantos et al., 2015; Sigrist et al., 2017). Acquired fibrinogen deficiency is often associated with consumptive coagulopathies (Raber, 1990). Hypofibrinogenemia may also reflect hyperfibrinolysis, where excessive degradation of fibrin compromises hemostasis (Cole et al., 2018; Sigrist et al., 2017, 2018, 2021). Fibrin is produced by thrombin-mediated cleavage of fibrinogen and crosslinked by activated factor XIII, while D-dimers are formed from the degradation of this crosslinked fibrin (Stokol et al., 2000). Elevated D-dimer levels, which therefore signal active fibrinolysis, are also observed in humans with thrombosis, cancer, or DIC. In experimentally infected foxes, *A. vasorum* D-dimer concentrations were increased - particularly in younger animals - alongside leukocytosis and thrombocytopenia (Webster et al., 2017). These findings mirror the current data and underscore how host age and the timing of infection can shape hematological responses. Given the heterogeneity of our clinical cases, including unknown infection durations, such factors likely contributed to the observed variability. Importantly, this variability reflects an inherent limitation of studies based on naturally infected animals. In contrast to experimental infections, the exact duration of infection, the worm burden, and the possibility of repeated exposure cannot be determined in clinical cases. These parameters are likely to substantially influence both the host immunopathological response and the measured hemostatic variables. It is therefore plausible that part of the variability observed in our dataset reflects differences in infection stage or parasite load rather than solely host-specific factors. To increase the number of evaluable cases, a small number of experimentally infected dogs were included in the cohort; however, the analysis was restricted to parameters directly comparable with those obtained from naturally infected animals, and experimental infection variables such as inoculation dose or infection timing were not considered.

Interestingly, fibrinogen levels tended to be higher in Inf_norm_ dogs than in UC. Based on our hematological, conventional coagulation, and ROTEM data, we propose the following progression: (i) infected animals may initially mount a compensatory response, increasing the production of clotting components such as fibrinogen and thrombocytes (as suggested by elevated thrombocrit and enhanced Fib-TEM and InTEM ROTEM MCF and MCE); (ii) over time, these resources become depleted, supporting a shift toward consumptive coagulopathy and, ultimately, DIC. DIC, often triggered by sepsis, is characterized by systemic hypercoagulability, excessive thrombin and fibrin formation, platelet activation, and widespread consumption of clotting factors (Ramsey et al., 1996). This leads to microvascular thrombosis, hemorrhage, and organ damage. We hypothesize that the differences observed between Inf_norm_ and Inf_hypo_ dogs reflect varying stages of infection and individual factors such as age, comorbidities, and genetic predispositions. Because infection duration and parasite burden cannot be retrospectively quantified in naturally infected dogs, these variables represent important uncontrolled factors in our study. A longer-standing infection or a higher number of adult worms interacting with the host through excretory/secretory (E/S) products could plausibly increase the risk of coagulation disturbances through cumulative inflammatory and endothelial effects. Future longitudinal studies using controlled experimental infections or well-characterized clinical cohorts will be required to directly test this hypothesis and disentangle infection stage from host susceptibility factors.

Despite increasing evidence, the underlying pathogenic mechanisms of coagulopathies in canine angiostrongylosis remain incompletely understood. While *A. vasorum* E/S proteins were proposed to drive hyperfibrinolysis, this could not be confirmed *in vitro*, suggesting additional *in vivo* mechanisms and potential roles for other parasite stages (Gillis-Germitsch et al., 2021a). At the protein level, the coagulation cascade appears disrupted, especially in chronic infection. In a previous time-course study in experimentally infected dogs, we observed reduced levels of key coagulation proteins, including mannan-binding serine proteases and factors V and XIIIB (Tritten et al., 2021), supporting both enhanced fibrinolysis and unstable fibrin clot formation. In contrast, experimentally infected foxes - who typically do not show bleeding - exhibited downregulation of different hemostatic proteins, including factors IX, X, XIIIA, and protein C, along with upregulation of others, reflecting a complex and dynamic modulation of coagulation pathways (Gillis-Germitsch et al., 2021b). To explore molecular mechanisms potentially underlying the hemostatic alterations observed in the present study, we analyzed host transcriptional responses associated with infection. Several pathways emerged that may mechanistically link inflammation, endothelial dysfunction, and coagulation disturbances. Our transcriptomic analysis complements these findings, and we highlight its most striking features.

(i) *Enhanced metallopeptidase activity and thrombo-inflammatory signaling.* One of the most prominent transcriptional signatures observed in infected dogs was increased expression of metallopeptidases. *MMP1*, *MMP8*, and *MMP9*, as well as *ACE2* and *CPA3*, were among the upregulated genes common to both infected groups. MMPs play key roles in extracellular matrix remodeling by degrading its components and regulating pro-inflammatory response cytokines, thus contributing to the inflammatory response (Lee and Kim, 2022). Over 20 vertebrate MMPs have been identified, which are grouped according to their substrates and structures. While MMPs perform various physiological functions, they have been most extensively studied in the context of angiogenesis. MMP1 and MMP9 are platelet-associated MMPs. MMP1 and MMP8 are collagenases, while MMP9 is a gelatinase that contains a fibronectin type II motif. Activation of monocytes by fibrinogen has been shown to increase MMP9 secretion, which, in turn, enhances monocyte aggregation through an autocrine mechanism. Pharmacological inhibition of MMP9 prolongs coagulation time, underscoring its significant role in coagulation (Kaneider et al., 2010). Both MMP1 and MMP9 cleave and inactivate the tissue factor pathway inhibitor (TFPI; see Fig. 3), a protease inhibitor of tissue factor-induced coagulation, with MMP9 demonstrating greater efficiency. In this context, upregulation of MMP9 would lead to enhanced coagulation. MMP9, generally increased in inflammation, functions in neutrophil recruitment and has been described in many helminth infections (Bruschi and Pinto, 2017). Importantly, the involvement of MMP9 (and MMP2) in the mechanisms responsible for organ damage (mainly the brain) in the infection caused by *A. cantonensis* in mice has been established, through tight junction protein disruption and Purkinje cell degeneration (Lee et al., 2004; Lai et al., 2020). MMP1 levels correlate with the presence of inflammatory cytokines; it plays a role in the development of deep vein thrombosis in human patients (Zhang et al., 2019). MMP1 was shown to cleave protease-activated receptor 1 (PAR1) in various disease contexts, including sepsis, which initiates platelet activation (Esmon, 2004; Heuberger and Schuepbach, 2019). Interestingly, MMP8 and MMP9 may also induce platelet activation *via* PAR1 (Lee et al., 2010). PAR1 is highly expressed in platelets and endothelial cells and is the prototypical receptor of thrombin; it plays a key role in mediating the interplay between coagulation and inflammation and is central to endothelial barrier integrity (Heuberger and Schuepbach, 2019). Accordingly, the autocrine MMP1-PAR1 signaling system on the vascular endothelium links inflammation, barrier function, coagulation, and sepsis outcomes (Tressel et al., 2011). MMP1 levels rise in the plasma of patients with severe sepsis, which may prove useful as a predictor of early mortality (Tressel et al., 2011). In mice, MMP1 acts as an agonist of PAR1 on endothelial cells, and MMP1 activity blockade suppressed DIC and lung vascular permeability, among others (Tressel et al., 2011). Taken together, these findings suggest that increased metallopeptidase activity may contribute to the thrombo-inflammatory conditions observed in *A. vasorum* infection. How a greater than 16-fold increase in *MMP1* transcript expression, as observed here, would translate into protein activity, remains to be investigated. We reported previously serum protein alterations in laboratory-infected dogs without overt signs of bleeding (Tritten et al., 2021). No MMP was found among significantly dysregulated proteins, but rather, other peptidases, such as Bone Morphogenic Protein 1 (BMP1), another collagenase, displayed elevated levels in late time points post-inoculation (starting at 75 days post-infection and showing an increasing trend over time). The various BMP1 functions are contextual and interconnected. Among others, it cleaves Thrombospondin-1 (Anastasi et al., 2020), which in turn promotes platelet adhesion. Collectively, MMP1, MMP8 and MMP9 promote platelet activation *via* collagen exposure and endothelial damage, which may amplify coagulation and thrombo-inflammatory responses, through extracellular matrix (ECM) degradation (Newby, 2008; Lu et al., 2011). The anti-fibrinolytic effects of MMPs are amplified by upregulated SERPINB2 (in both infected groups *vs* UC), which inhibits fibrinolysis by interacting with tPA and uPA, thereby promoting clot stability (Ritchie et al., 2000; Medcalf, 2011).
  ACE2 is expressed in type II alveolar cells and platelets and plays an important role in maintaining pulmonary tissue integrity and vascular homeostasis through regulation of the renin-angiotensin system (Imai et al., 2005; Kuba et al., 2005; Wang et al., 2020); accordingly, circulating *ACE2* transcripts may also reflect epithelial or endothelial injury (Zhang et al., 2020; Jalaleddine et al., 2023; Li et al., 2024). Moreover, ACE2 exerts antithrombotic effects by degrading Angiotensin II (Ang2), thereby preventing its procoagulant stimulation of PAI-1. Illustrating the organ-specific roles of ACE2, its interaction with SARS-CoV-2 on platelets has been proposed to contribute to thrombotic complications in COVID-19 (Zhang et al., 2020).
  Overall, metallopeptidases were similarly upregulated in both Inf_norm_ and Inf_hypo_ groups, except for ACE2, which was strongly upregulated in Inf_hypo_ only (log_2_ ratio: 6.36). This suggests they represent a general response to infection, generally promoting coagulation.
(ii) *Compromised endothelial integrity.* Several transcriptional alterations observed in infected dogs point toward disruption of endothelial signaling pathways that regulate vascular integrity and coagulation. Among the downregulated genes in Inf_norm_ *vs* UC, Notch3 is critical to vascular health (see Fig. 3). Notch3-deficient adult mice show phenotypes in brain and retinal vasculature, resulting from vascular smooth muscle cell degeneration and loss through apoptosis (Belin de Chantemèle et al., 2008). This causes loss of vessel integrity, hemorrhage and loss of blood-brain barrier function (Hosseini-Alghaderi and Baron, 2020). Similarly, FARP1 (significantly downregulated in Inf_norm_ *vs* UC) was identified as a novel regulator of endothelial barrier integrity, where silencing FARP1 (along with the interactor CDC42) contributed to a strong negative effect on endothelial barrier function and cell morphology (Amado-Azevedo et al., 2017). Retinoic acid-induced protein 3 (downregulated in Inf_norm_ *vs* UC), a protein encoded by the gene *GPRC5A* (a.k.a. *RAIG1*, *RAI3*), has several putative functions in maintaining homeostasis of epithelial cells, among others. Decreased *GPRC5A* was also observed in the context of lung cancer development associated with lung inflammation (Fujimoto et al., 2012). In mice, *GPRC5A* deficiency leads to an increased inflammatory response in lung tissues (Liao et al., 2015). The underlying mechanisms remain unresolved to date and may involve interactions between AHRR (downregulated in all infected groups) with other signal transduction pathways, including C/EBP and NF-κB, leading to the regulation of specific inflammatory cytokines (Vogel et al., 2016). AHRR inhibits both induced and constitutive aryl hydrocarbon receptor (AHR) transcriptional activity by competing with AHR for aryl hydrocarbon nuclear translocator. In a negative feedback loop, AHR induces the transcriptional upregulation of AHRR, which subsequently mediates suppression of AHR activity (Hahn et al., 2009). Shear stress and some toxins (indoxyl sulfate) activate AHR through different pathways, which in turn increase endothelial tissue factor (TF, the main initiator of coagulation) expression in an AHR-dependent manner (Lano et al., 2020). This suggests that altered AHRR-dependent AHR regulation may have consequences for coagulation. TIE2 is a receptor for angiopoietins mainly expressed by endothelial cells and is associated with vascular health; TIE2 mutations cause morphological changes in endothelial cells following activation of the mitogen-activated protein kinase (MAPK) pathway and depletion of extracellular matrix fibronectin. TIE2 (upregulated in Inf_hypo_ *vs* UC) also induces the plasminogen/plasmin proteolytic system, leading to coagulopathy reflected by high D-dimer levels (Nätynki et al., 2015). TIE2 signaling has been known to decline in sepsis, and genetically reduced TIE2 alone is sufficient to mimic the excessive fibrin deposition in sepsis (Higgins et al., 2018). In a mouse model, where lipopolysaccharide was used to induce DIC, it was shown that disruption of the endothelial TIE2 axis is a preceding sign, sentinel event, of overt septic DIC (Higgins et al., 2018). Proteomics in septic DIC patients identified a network interweaving vascular function, endothelial inflammation, and coagulation around the TIE2 antagonist, angiopoietin 2 (ANGPT-2), which was strongly associated with traditional DIC markers, including platelet counts (Higgins et al., 2018). A fraction of monocytes express TIE2 (2–5% of bone marrow cells in healthy mice) as well (De Palma et al., 2005); however, they are deemed unlikely to account for fibrin deposition (Higgins et al., 2018). Normalization of TIE2 activation through administration of ANGPT-1 *via* gene transfer efficiently counteracted DIC to normalize the thrombotic response. These findings suggest that *A. vasorum* infection is associated with disruption of endothelial homeostasis and vascular integrity. The mechanisms underlying this effect remain unclear and may involve both parasite-associated vascular damage and host-mediated inflammatory or immunomodulatory processes. Altered expression of key regulators such as Notch3, FARP1, GPRC5A, AHRR, and TIE2 points to endothelial dysfunction as a central feature of infection. This vascular impairment likely contributes to coagulopathy, linking inflammation, endothelial activation, and fibrinolysis.
(iii) *Impaired pathogen recognition* - particularly of pathogenic glycoproteins - was evident among the downregulated transcripts in infected dogs. Both macrophage mannose receptor C-type 1 (*MRC1/CD206*) and receptor-type tyrosine-protein phosphatase S (*PTPRS*), previously implicated in helminth infections, were downregulated in both infected groups in this study. CD206 is expressed by (alternatively-activated) macrophages and dendritic cells and likely binds to glycosylated E/S products released by helminths; its exact role, however, remains uncertain (Rolot and Dewals, 2018). In the human immune system, PTPRS represents a conserved plasmacystoid dendritic cell (pDC)-specific inhibitory receptor, necessary to limit spontaneous interferon (IFN) production and immune-mediated intestinal inflammation (Bunin et al., 2015). The expression of PTPRS is higher on the surface of naive pDCs but is reduced upon cell activation; PTPRS reduction seems to be necessary for cytokine production by activated pDCs (Bunin et al., 2015). Interestingly, PTPRS is a signature of pre-DCs in dogs, while STYXL2 - another downregulated transcript in *A. vasorum*-infected dogs - is a signature of canine pDCs (Ammons et al., 2023). STYXL2 (DUSP27) is a poorly characterized pseudo-phosphatase, whose function in an immune context remains to be described. Secretory Carrier Membrane Protein 5 (SCAMP5) colocalizes with IFNα in activated human pDCs, supporting a role of this carrier in the secretion of type I IFN by pDCs (Pouw et al., 2022). Cytochrome P450 is commonly downregulated in the context of acute inflammation (Stavropoulou et al., 2018). CYP2D15 is the canine orthologue of the human CYP2D6 (the second most abundant human CYP450). Together, these findings suggest that *A. vasorum* infection may impair dendritic cell function and disrupt early innate immune signaling, potentially dampening type I interferon responses and promoting parasite persistence.

Study limitations: this study has several limitations that should be considered when interpreting the results. First, the study population consisted of naturally infected dogs, which precludes precise knowledge of infection duration, parasite burden, and the potential occurrence of repeated infections. These factors likely influence both the immunopathogenic mechanisms of *A. vasorum* infection and the resulting hemostatic abnormalities. Secondly, although we minimized treatment-related confounding by excluding dogs that had received treatments potentially interfering with coagulation within four weeks prior to presentation, other clinical variables inherent to retrospective case recruitment cannot be fully mitigated. Finally, the sample sizes in both *A. vasorum*-infected groups were small, which limits the statistical power of some comparisons. The absence of distinct transcriptomic signatures associated with coagulation status may therefore reflect limited statistical power and individual variability among infected dogs, including differences in immune responses and infection-related factors that cannot be controlled in naturally infected clinical cohorts. Despite these limitations, the differences observed between infected dogs with and without coagulopathy, together with complementary transcriptomic and ROTEM data, provide valuable insight into the complex mechanisms underlying coagulopathies in canine angiostrongylosis.

## 5 Conclusions

The multifaceted alterations observed in hematologic, coagulation, transcriptomic, and proteomic profiles of *A. vasorum*-infected dogs point to a dynamic and evolving disruption of hemostatic balance. Importantly, the clinical and hemostatic findings together suggest that angiostrongylosis is characterized by a continuum of coagulation disturbances rather than a single defined coagulopathy. While some animals show signs of early compensatory hypercoagulability, others progress toward a state consistent with DIC, marked by factor consumption, endothelial dysfunction, and impaired fibrinogen-dependent clotting. The factors determining this transition remain incompletely understood but likely include infection stage, host inflammatory responses, and the extent of endothelial injury. In previous clinical analyses of these cohorts, dogs presenting with hypofibrinogenemia and hyperfibrinolysis were more likely to exhibit clinical bleeding (Sigrist et al., 2021). Concurrent upregulation of metallopeptidases (e.g. MMP9), along with reduced fibrinogen and elevated D-dimers, supports a role for hyperfibrinolysis, particularly in more severely affected animals. The observed increase in anti-fibrinolytic effectors (e.g. SERPINB2) may reflect a compensatory response to excessive fibrinolytic activity. This evolving balance between procoagulant activation, endothelial injury, and fibrinolysis likely explains why clinical presentation does not always correlate directly with measured hemostatic parameters. These findings suggest that *A. vasorum* infection drives a spectrum of thrombo-inflammatory responses shaped by infection stage, immune status, and vascular injury, ultimately converging on a mixed phenotype of consumptive coagulopathy with secondary hyperfibrinolysis.

## Ethical approval

All applicable international and institutional guidelines for the care and use of animals were followed: material from naturally infected animals was obtained in the frame of diagnostic analyses, and data from experimentally infected animals had been obtained in previously ethically approved studies. Informed owner consent was obtained for both healthy and infected dogs (experimental animal trial no. 2014_40E_FR). All clinical parameters were assessed using tools and procedures established at the University Veterinary Hospital of the University of Zurich following standard procedures and are routinely used in clinical patients.

## CRediT authorship contribution statement

**Lucienne Tritten:** Conceptualization, Funding acquisition, Investigation, Formal analysis, Writing - original draft, Writing - review & editing. **Annageldi Tayyrov:** Investigation, Writing - review & editing. **Lennart Opitz:** Conceptualization, Investigation, Formal analysis, Writing - review & editing. **Annette P.N. Kutter:** Conceptualization, Formal analysis, Writing - review & editing. **Nadja E. Sigrist:** Conceptualization, Formal analysis, Writing - review & editing. **Natalie Hofer-Inteeworn:** Formal analysis, Writing - review & editing. **Claudia Kümmerle-Fraune:** Formal analysis, Writing - review & editing. **Manuela Schnyder:** Conceptualization, Supervision, Funding acquisition, Formal analysis, Writing - review & editing.

## Funding

We thank the Albert Heim-Stiftung for funding of this study (project no. 121). This study was also financed by institutional funds.

## Declaration of competing interests

The authors declare that they have no known competing financial interests or personal relationships that could have appeared to influence the work reported in this paper.

## Data Availability

All data generated or analyzed during this study are included in this published article and its supplementary files. The RNA-seq data, are available at NCBI Sequence Read Archive under the accession number PRJEB53546.

## Appendix A Supplementary data

**Supplementary file 1:**Supplementary Fig. S1**.** Hematology data presented by study groups. Multimedia component. 1

**Supplementary file 2:**Supplementary Table S1**.** Blood work. Blood cell counts, coagulation, and ROTEM data for each sample. Supplementary Table S2. Transcriptome analysis_AllInfected_over_UC. No FDR or *P*-value cut-off is applied. Supplementary Table S3. Transcriptome analysis_Inf_norm__over_UC. No FDR or *P*-value cut-off is applied. Supplementary Table S4. Transcriptome analysis_Inf_hypo__over_UC. No FDR or *P*-value cut-off is applied. Supplementary Table S5. Transcriptome analysis_Inf_hypo__over_Inf_norm_. No FDR or *P*-value cut-off is applied. Supplementary Table S6. Gene Set Enrichment Analysis_ All Infected_over_UC. Supplementary Table S7. Gene Set Enrichment Analysis_ Inf_norm__over_UC. Supplementary Table S8. Gene Set Enrichment Analysis_ Inf_hypo__over_UC. Multimedia component. 2
