# Supplementary material for: Increased metallopeptidase activity, compromised endothelial integrity and impaired pathogen recognition revealed by transcriptomics in Angiostrongylus vasorum-infected dogs with hypocoagulability and vascular dysfunction
Source: Curr Res Parasitol Vector Borne Dis. 2026 Apr 12;9:100378. doi: 10.1016/j.crpvbd.2026.100378 (PMC13185918; doi:10.1016/j.crpvbd.2026.100378)
Supplement: Supplementary file 1 — Supplementary Fig. S1. Hematology data presented by study groups. Multimedia component. 1 [file mmc1.pdf]

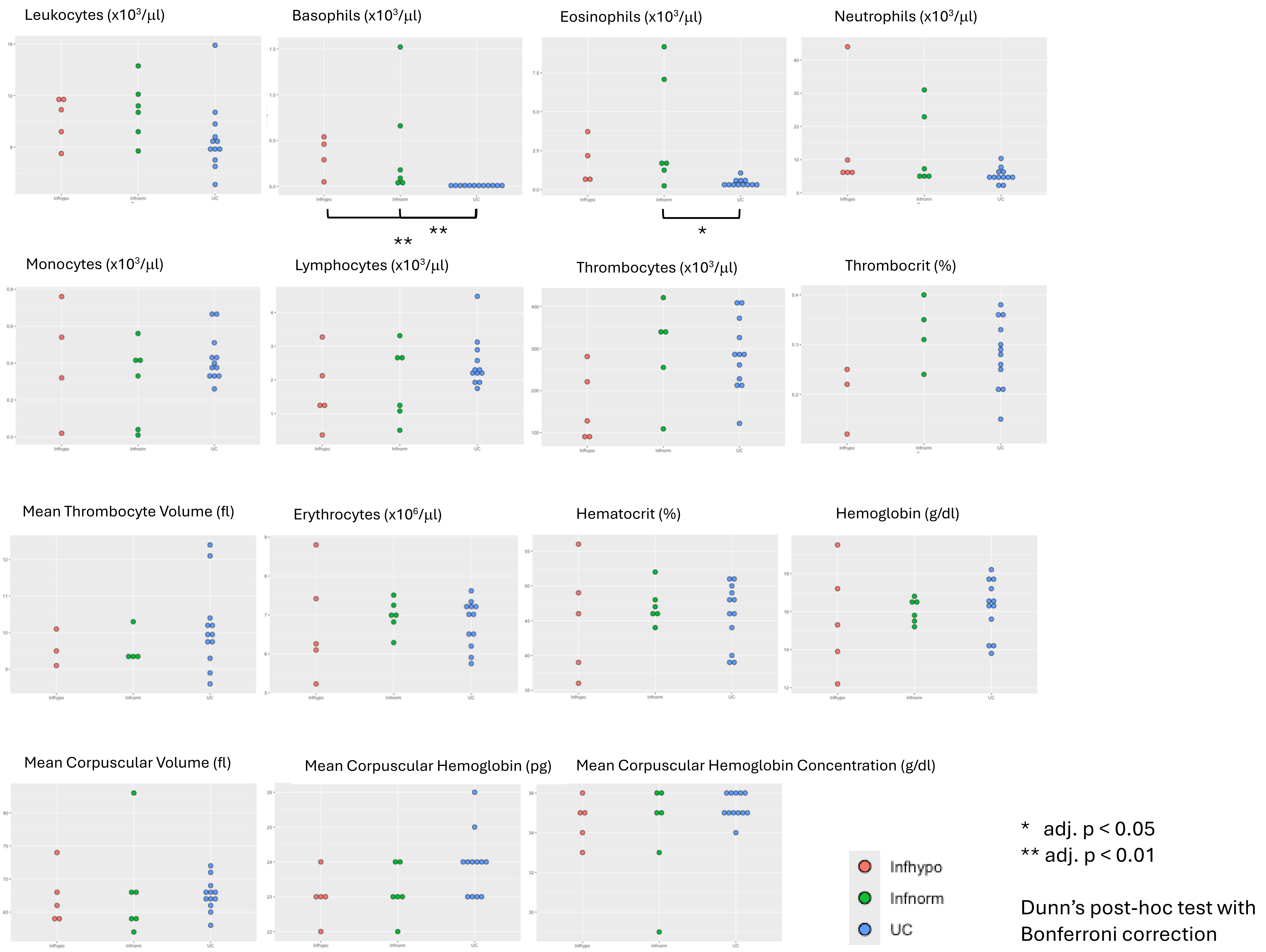

Supplementary Figure S1. Hematology data presented by study groups. *Abbreviations*: Infhypo, Infected hypocoagulable; Infnorm, Infected normocoagulable; UC, uninfected controls; adj. p, adjusted *P* value.
